# Associated Veterinary Faculties

**Published:** 1895-05

**Authors:** 


ASSOCIATION VETERINARY FACULTIES NORTH AMERICA.—Perhaps it is not too early to suggest that one of the future questions to be confronted by this all-important veterinary body shall be as to its position relative to the State statutes now being engrafted upon the laws of various States creating Boards of Veterinary Medical Examiners that shall vouchsafe to their people, those who are well and sufficiently educated, to return to their employers a just recompense and return when employed and when intrusted with valuable property whose existence is endangered by disease and other property imperiled by its presence.

